# Fish bone–induced liver abscess secondary to duodenal perforation: a case report

**DOI:** 10.3389/fmed.2026.1806769

**Published:** 2026-03-17

**Authors:** Zhengjian Wang, Zhe Wang, Xuda Ji, Chaoqun Ma, Yinlong Xu, Hong Chang, Fang Liu, Fangfeng Liu

**Affiliations:** 1 Department of Hepatobiliary Surgery, Shandong Provincial Hospital Affiliated to Shandong First Medical University, Jinan, Shandong, China; 2 Shandong Provincial Hospital, Shandong University, Jinan, Shandong, China; 3 Department of Radiology, Shandong Provincial Hospital Affiliated to Shandong First Medical University, Jinan, Shandong, China

**Keywords:** duodenal perforation, fish bone, laparoscopy, liver abscess, migrated foreign body

## Abstract

**Introduction:**

Gastrointestinal perforation caused by ingested foreign bodies typically presents with acute abdominal symptoms. However, migration of a fish bone through the posterior wall of the duodenum into the liver, resulting in liver abscess formation without abdominal pain, is extremely rare and easily misdiagnosed. This case highlights an atypical clinical presentation and important diagnostic considerations.

**Case description:**

A 56-year-old man presented with recurrent fever for 25 days without abdominal pain or gastrointestinal symptoms. Laboratory tests revealed elevated inflammatory markers. Contrast-enhanced computed tomography demonstrated a liver abscess in segment V and a linear hyperdense structure extending from the duodenum into the hepatic parenchyma, suggesting foreign body migration. Emergency laparoscopic exploration was performed, during which a migrated fish bone was removed, the liver abscess was drained, and the duodenal perforation was repaired. The postoperative course was uneventful, with normalization of body temperature on the first postoperative day. The patient was discharged on postoperative day four and showed no recurrence during follow-up.

**Conclusion:**

This case emphasizes that gastrointestinal foreign body perforation and migration should be considered in patients with liver abscess and fever of unknown origin, even in the absence of abdominal pain. Detailed dietary history-taking and careful interpretation of CT imaging are essential for accurate diagnosis, and laparoscopic surgery provides an effective diagnostic and therapeutic approach.

## 1 Introduction

Ingested sharp foreign bodies, particularly fish bones, represent a common reason for presentation to emergency departments and gastrointestinal endoscopy units worldwide (1–5). Previous studies have shown that approximately 80%–90% of ingested foreign bodies pass spontaneously through the gastrointestinal tract, while only 10%–20% require endoscopic intervention; the proportion of cases complicated by gastrointestinal perforation or requiring surgical management is generally <1% (6, 7). Typical foreign body–related perforations most frequently occur at anatomical narrowing or vulnerable sites, such as the physiological constrictions of the esophagus, the gastric antrum, or the ileocecal region, and usually present with acute abdominal pain and signs of peritoneal irritation, making the diagnosis relatively straightforward (8). In contrast, when a foreign body penetrates the duodenum—particularly the posterior wall, which is located in the retroperitoneal space—the clinical presentation often deviates markedly from this classic pattern (8, 9).

Perforation of the posterior duodenal wall may lack typical signs of an acute abdomen because of the buffering effect of the retroperitoneal space, resulting in insidious or occult early manifestations. Even more rarely, under the combined influence of chronic inflammation, fibrous encapsulation, and relative visceral motion, a sharp foreign body may undergo secondary migration (10, 11). Among these scenarios, migration into the adjacent hepatic parenchyma with subsequent liver abscess formation is one of the least frequently reported and most diagnostically challenging entities. Systematic reviews and case series indicate that migratory liver abscesses caused by fish bones or other foreign bodies account for only a very small fraction of all pyogenic liver abscesses (12–17). Overall, this condition remains uncommon, with only limited case reports described in the literature, and its true incidence is likely underestimated.

The present case exemplifies this diagnostic dilemma. The patient presented with isolated fever of unknown origin lasting nearly 4 weeks as the sole complaint, without abdominal pain, nausea, vomiting, or other gastrointestinal symptoms. On admission, physical examination revealed no abdominal tenderness, rebound tenderness, or muscular guarding, and there were no signs of peritoneal irritation. Laboratory investigations demonstrated markedly elevated inflammatory markers, whereas liver function tests and pancreatic enzyme levels were largely within normal ranges. Such nonspecific clinical features can easily be misinterpreted as those of a simple infectious liver abscess or other inflammatory conditions, leading to delayed recognition of an underlying foreign body–related etiology. Given that retained foreign bodies may not only result in persistent or refractory infection but also pose risks of vascular erosion or extension to adjacent structures (18–21), early identification of the cause and timely definitive treatment are of critical importance.

Accordingly, we report a case of duodenal fish bone migration leading to liver abscess formation, presenting with isolated fever as the initial manifestation. This report highlights the atypical clinical presentation, key imaging clues, and the integrated diagnostic and therapeutic value of laparoscopic management. Through this case, we aim to enhance clinicians’ awareness of the disease spectrum of painless gastrointestinal perforation with foreign body migration and to promote a diagnostic approach that integrates unexplained fever and liver abscess as entry points, combined with meticulous dietary history-taking and careful interpretation of computed tomography findings, thereby reducing diagnostic delay and improving patient outcomes.

## 2 Case presentation

### 2.1 Diagnostic assessment

A 56-year-old male was admitted to our hospital with a chief complaint of “recurrent fever persisting for 25 days.” Approximately 25 days prior to admission, the patient had a history of accidental fish bone ingestion. Subsequently, he developed an unexplained, persistent low-grade fever with a peak temperature of 38 °C. Notably, throughout this period, he denied any gastrointestinal symptoms such as abdominal pain, nausea, or vomiting. He was initially treated at a local clinic with penicillin-based antibiotics (specific agents unknown) for approximately 1 week, but the fever remained uncontrolled.

Upon admission, the patient presented with a temperature of 37.6 °C. Vital signs revealed a heart rate of 76 beats/min, blood pressure of 122/79 mmHg, respiratory rate of 14 breaths/min, and oxygen saturation of 98% on room air. From admission until surgery, his temperature fluctuated between 37.0 °C and 38.0 °C, with the highest recorded value of 38.0 °C occurring on the second hospital day. Symptomatic antipyretic therapy with lysine acetylsalicylate was administered. On the morning of surgery, his body temperature was 36.8 °C. The patient denied chills or rigors, and no clinical signs of systemic infection were observed. He was alert and in no acute distress, with no jaundice or skin rash. Cardiopulmonary examination was normal, with clear breath sounds and regular cardiac rhythm. No peripheral edema or focal neurological deficits were noted. Abdominal examination revealed a soft abdomen without tenderness, rebound tenderness, or guarding; Murphy’s sign was negative. Laboratory investigations indicated an inflammatory response. The white blood cell count was 9.35 × 10^9^/L with an elevated neutrophil percentage of 76.3%, and interleukin-6 (IL-6) was 72.2 pg./mL. The platelet count was 235 × 10^9^/L, and procalcitonin was 0.2 ng/mL. Liver biochemistry showed AST 19 U/L, ALT 26 U/L, ALP 77 U/L, and GGT 248 U/L. Total bilirubin was 15.7 μmol/L, albumin was 35.5 g/L, and the international normalized ratio (INR) was 1.12. Renal function was preserved, with serum creatinine 80.3 μmol/L, uric acid 318 μmol/L, and estimated glomerular filtration rate (eGFR) 105.3 mL/min/1.73 m^2^. C-reactive protein and erythrocyte sedimentation rate were not assessed at admission. Tumor markers (AFP, CEA, CA19-9) and Influenza A/B antigen tests were negative, ruling out common neoplastic diseases or specific viral infections. The patient had a 3-year history of coronary artery disease and had undergone right coronary artery stenting; he was on long-term therapy with ticagrelor, rosuvastatin, and metoprolol. He denied any history of diabetes, chronic liver disease, or immunodeficiency.

**Figure 1:**
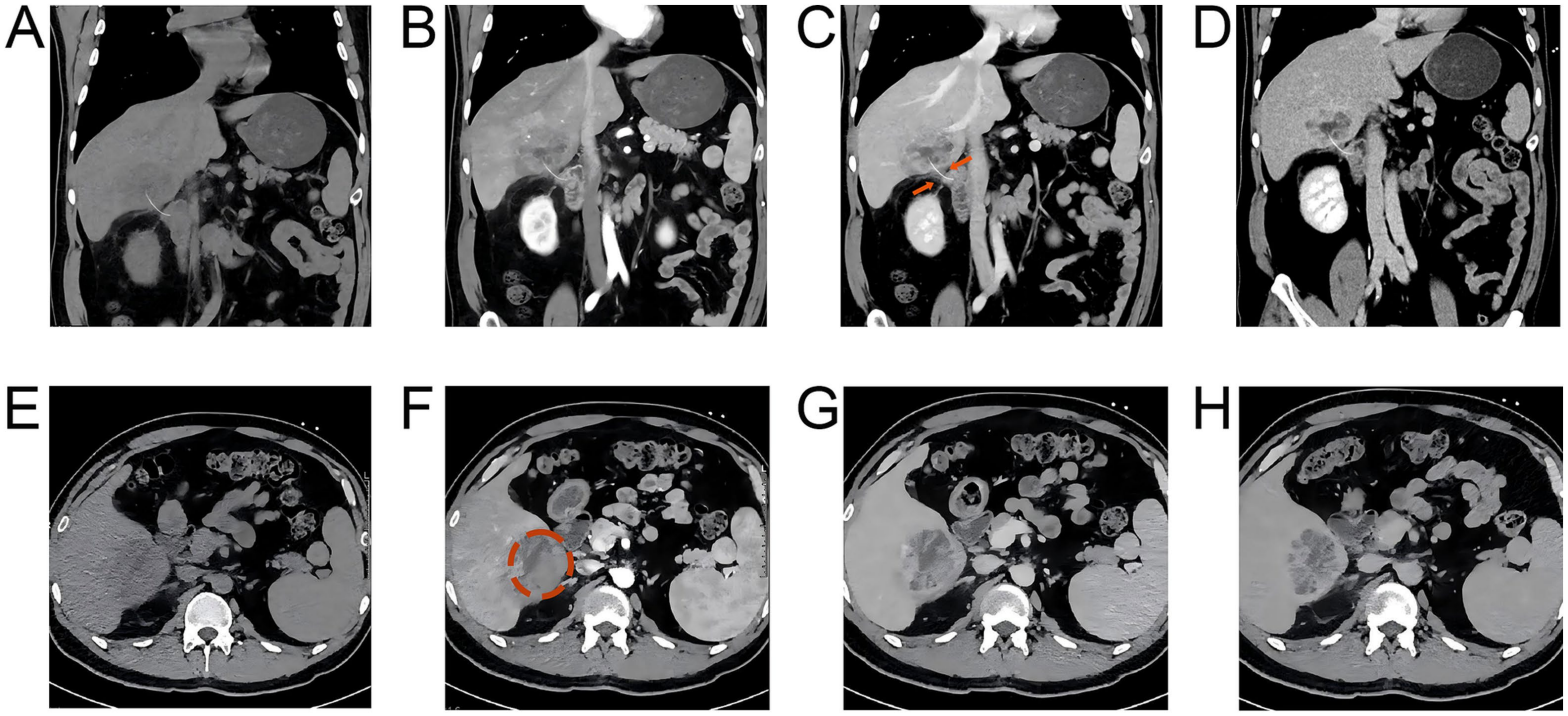
Computed tomography (CT) scan images. **(A)** Non-contrast coronal view: an arc-shaped linear hyperdense lesion (foreign body, fish bone) was identified with its two ends located in the right hepatic lobe and the descending duodenum, respectively. A corresponding massive hypodense lesion (hepatic abscess) was observed in the right hepatic lobe. Mild exudative changes were noted in the space between the descending duodenum and the right hepatic lobe. **(B)** Arterial phase coronal view: the hepatic abscess in the right hepatic lobe showed heterogeneous marked enhancement. An irregular hypodense area was visualized within the abscess, predominantly around the foreign body. Extensive hyperenhancement was detected in the hepatic parenchyma surrounding the abscess. **(C)** Portal venous phase coronal view: the hepatic abscess in the right hepatic lobe exhibited enhancement of the thick-walled septa. No significant enhancement was observed in the irregular hypodense area inside the abscess. The upper orange arrow indicates the hyperdense foreign body, and the lower orange arrow indicates the incompletely formed sinus tract surrounding the foreign body and associated periduodenal inflammatory changes. **(D)** Hepatic parenchymal phase coronal view: the thick-walled septa of the hepatic abscess in the right hepatic lobe showed density similar to the surrounding hepatic parenchyma, while no obvious enhancement was noted in the abscess cavity. **(E–H)** Non-contrast axial views, non-contrast and dynamic contrast-enhanced images: a hypodense lesion was found in the right hepatic lobe on the non-contrast scan. The lesion presented progressive enhancement of the thick wall and multilocular septa on contrast-enhanced scans, with no significant enhancement in the internal abscess cavity. The abscess cavity is indicated by the orange circle. **(F)** Extensive hyperenhancement was shown in the hepatic parenchyma surrounding the lesion.

Imaging provided critical clues for the diagnosis. Abdominal contrast-enhanced computed tomography (CT) revealed an irregular hypodense lesion measuring approximately 4.5 × 3.0 cm in segment V of the right hepatic lobe (Figure 1F). The lesion showed mild marginal enhancement in the arterial phase and continuous rim enhancement in the portal venous and delayed phases, consistent with the typical radiological features of a liver abscess. More importantly, a linear hyperdense shadow measuring approximately 3.5 cm in length and 2 mm in width (CT value ~280 HU) was identified adjacent to the abscess (Figure 1B, arrow). Proximally, it originated from the lateral wall at the junction between the duodenal bulb and the descending portion of the duodenum; distally, it traversed obliquely upward through hepatic segment V, terminating directly into the abscess cavity. This formed a clearly visible sinus tract suggestive of foreign body migration. The serosal surface of the lateral wall at the junction of the duodenal bulb and descending part appeared rough, accompanied by periduodenal fat stranding and flocculent exudation. Several reactive enlarged lymph nodes were also noted, indicating local chronic inflammation (Figures 1A–H). In light of this imaging diagnosis, a more detailed history was subsequently obtained, at which time the patient recalled a prior episode of accidental fish bone ingestion approximately 25 days earlier, which he had not initially considered relevant. This newly disclosed history provided a crucial etiological clue.

Based on the clinical presentation and imaging findings, a preliminary diagnosis was made: liver abscess secondary to a duodenal foreign body (fish bone) perforation and migration. To confirm the diagnosis and eradicate the infection source, an emergency laparoscopic exploration was planned for foreign body retrieval and abscess drainage.

### 2.2 Therapeutic intervention

Upon admission, the patient was febrile and had received approximately 7 days of penicillin-based antibiotic therapy at a community hospital without significant clinical improvement. Blood cultures were obtained immediately prior to initiation of further antimicrobial therapy. After contrast-enhanced CT established the preliminary diagnosis of liver abscess secondary to fish-bone perforation and migration, empirical intravenous antibiotic therapy was escalated while awaiting culture results. Ceftriaxone (1 g once daily) combined with metronidazole (0.5 g once daily) was administered to provide broader coverage against enteric gram-negative organisms and anaerobic bacteria.

Although the patient did not exhibit high-grade fever, chills, or signs of generalized peritonitis, the presence of a retained foreign body and a patent sinus tract between the duodenum and liver raised concern for ongoing infection and potential abscess rupture. Given that the infection appeared relatively localized and the preoperative laboratory parameters were stable, our team considered early definitive surgical source control to be the most appropriate strategy.

Regarding perioperative antiplatelet management, the patient had a history of coronary artery stenting and was receiving long-term ticagrelor therapy, which posed a potential risk of intraoperative bleeding. Ticagrelor was withheld for 2 days prior to surgery after multidisciplinary evaluation, and no bridging therapy was administered. The preoperative platelet count was 235 × 10^9^/L. The team proceeded with prompt surgical intervention without unnecessary delay.

**Figure 2:**
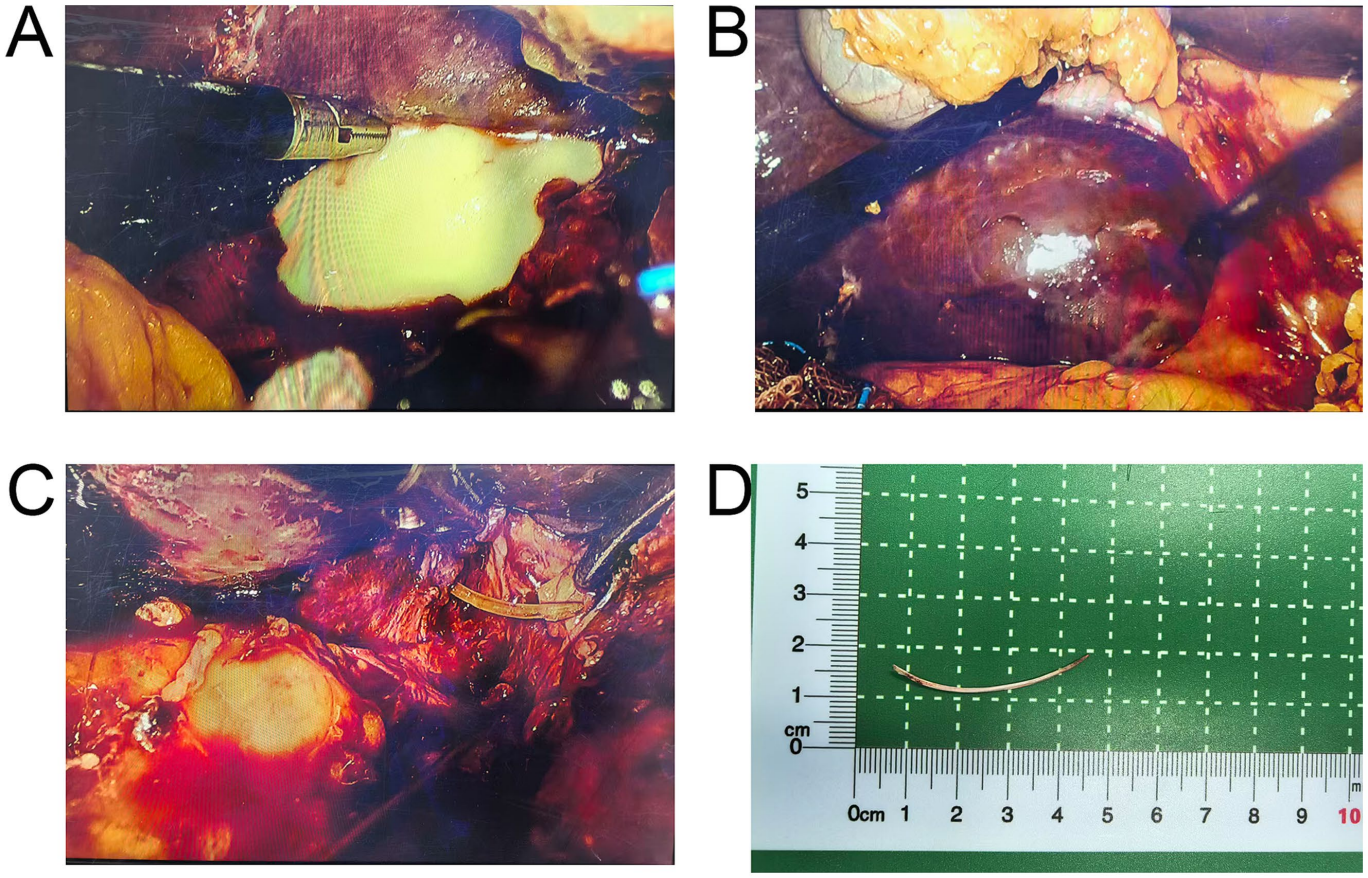
Intraoperative images and foreign body. **(A)** A large amount of pus accumulated in the hepatorenal space. **(B)** An ill-defined inflammatory mass on segment V of the liver surface. **(C)** The sharp tip of the fish bone perforating the hepatic parenchyma. **(D)** Gross specimen of the extracted fish bone.

Intraoperatively, extensive dense adhesions were observed between hepatic segment V, the transverse colon, the greater omentum, and the descending duodenum. After careful blunt dissection of the adhesions, the hepatorenal space was exposed, revealing a significant accumulation of milky-white purulent fluid (Figure 2A). The infection appeared relatively confined, with no evidence of diffuse peritoneal contamination. Exploration continued by tracing the source of the pus. A fibrous sinus tract was identified in the retroperitoneum at the posterior aspect of the junction between the duodenal bulb and the descending part, extending toward the liver. Following this tract to the liver surface, a hard, tough mass was palpable on segment V (Figure 2B). The fibrous capsule on the liver surface was incised to enter the abscess cavity, releasing a large amount of pus and necrotic tissue. On the medial wall of the cavity, a hard, sharp foreign body tip was found protruding from the hepatic parenchyma (Figure 2C). The object was carefully grasped and slowly extracted along its long axis, revealing a complete arc-shaped fish bone approximately 4.0 cm in length (Figure 2D), slightly longer than the preoperative CT estimate (~3.5 cm), which may be attributed to CT underestimation related to oblique orientation and partial volume effects.

Following extraction, a surge of pus drained from the intrahepatic tract. The abscess cavity was thoroughly debrided to remove necrotic tissue and repeatedly irrigated with diluted iodophor solution and saline until the fluid ran clear. Inspection of the original perforation site on the duodenum revealed only a pinpoint micro-perforation, which had been encapsulated by chronic inflammatory fibrosis; therefore, no omental patch was required. This was reinforced with interrupted sutures using 4–0 Prolene through the seromuscular layer, and given the tiny, well-encapsulated perforation, a leak test was deemed unnecessary. Finally, drainage tubes were placed in the cleaned liver abscess cavity and the hepatorenal space, respectively.

### 2.3 Outcome and follow-up

The surgery was uneventful, with successful removal of the foreign body and complete clearance of the abscess. The patient recovered well postoperatively. Mild postoperative fever was observed on the night of surgery (37.8 °C) and the morning of postoperative day 1 (37.6 °C), which was considered consistent with transient postoperative inflammatory response. Symptomatic antipyretic treatment with lysine acetylsalicylate was administered. By the afternoon of postoperative day 1, the body temperature had decreased to 36.6 °C and remained stable thereafter. Blood cultures had been obtained at admission prior to escalation of antimicrobial therapy. Final results became available postoperatively after 5 days of incubation (both aerobic and anaerobic), showing no bacterial growth. Intraoperative pus Gram staining demonstrated gram-negative bacilli and gram-positive cocci. Subsequent culture of the abscess fluid grew *Escherichia coli*, *Streptococcus anginosus group*, and *Bacteroides fragilis*, consistent with a gastrointestinal-origin polymicrobial infection. These organisms were well covered by the empirical regimen of ceftriaxone plus metronidazole. In accordance with the principle of avoiding unnecessary antibiotic escalation, the antimicrobial therapy was not modified. As the fever resolved and the clinical condition improved rapidly, antimicrobial therapy was discontinued after 3 days of postoperative ceftriaxone plus metronidazole therapy.

The abdominal drains functioned well, with drainage volume decreasing daily and the fluid transitioning from turbid to clear. During the first three postoperative days, no hemorrhagic drainage was observed, indicating resolution of bleeding risk. On postoperative day 3, the hepatorenal space drain was removed. A follow-up abdominal ultrasound performed the same day demonstrated marked shrinkage of the abscess cavity, permitting removal of the intrahepatic drain. After confirmation of stable hemostasis and absence of bleeding, ticagrelor was resumed at the preoperative oral dosage. The patient was discharged cured on postoperative day 4.

**Figure 3:**
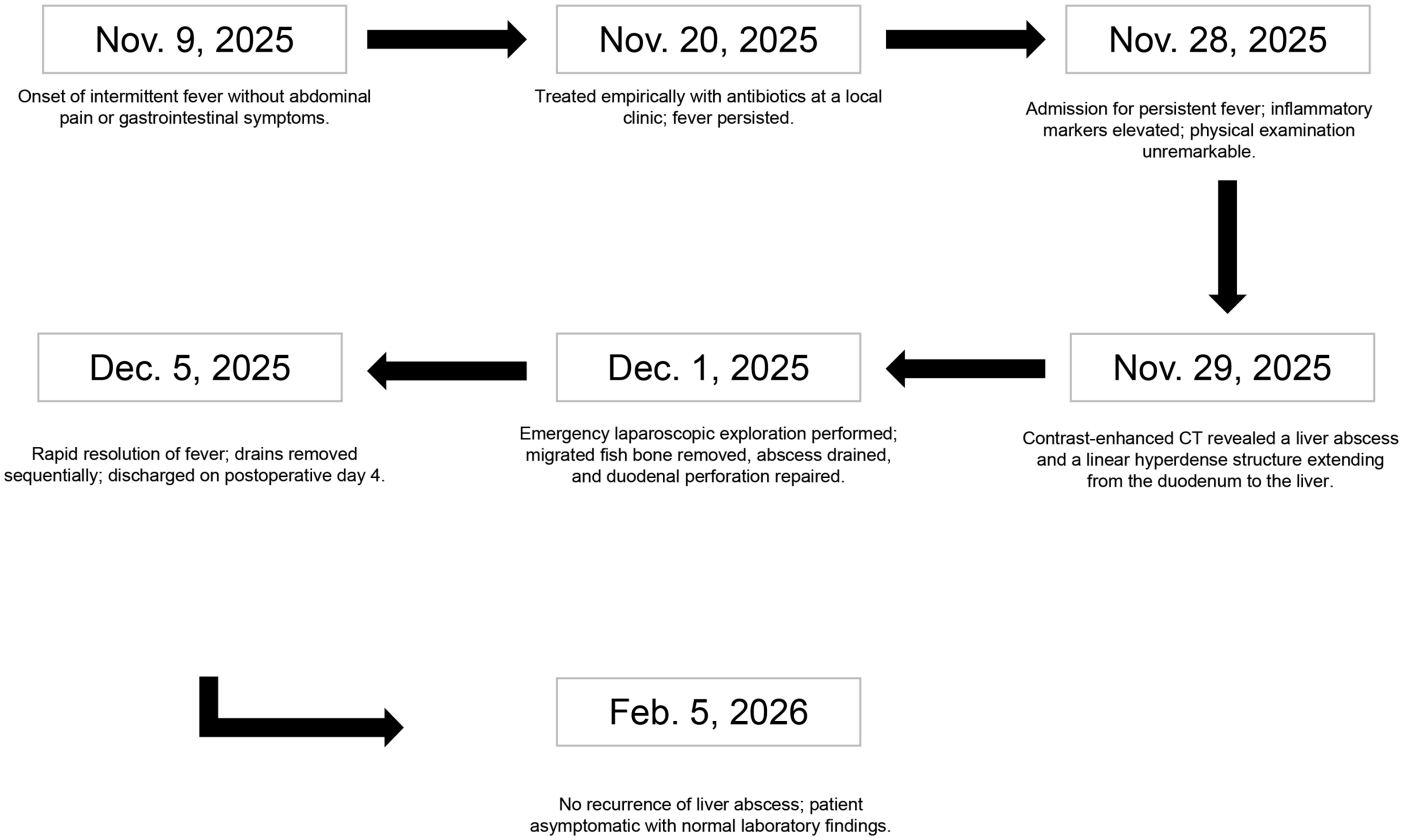
Timeline.

Regular follow-up after discharge showed no abnormalities. At the 3-month follow-up visit, the patient remained in good general condition with no recurrence of fever or other symptoms. Abdominal ultrasound revealed no residual abscess or recurrence, and liver function tests were normal. To date, the patient has shown no sequelae, indicating complete resolution of the infection and successful source control. Figure 3 chronologically summarizes the patient’s major symptoms, diagnostic findings, interventions, and outcomes.

## 3 Discussion

This case details a hepatic abscess secondary to duodenal fish bone migration presenting solely as isolated fever. Beyond its inherent rarity, the primary value of this report lies in its challenge to conventional diagnostic paradigms and the provision of a systematic management framework for such occult pathologies.

### 3.1 The insidious clinical presentation and diagnostic challenges

The most striking feature of this case was its clinical silence—specifically, the absence of abdominal pain and peritoneal signs (18). This presentation fundamentally disrupts the classic paradigm of gastrointestinal perforation, which is typically anchored in abdominal pain and peritonitis. This phenomenon is rooted in a unique anatomical basis: the posterior wall of the duodenum is a retroperitoneal structure. Perforations in this location are effectively buffered or contained by the retroperitoneal space, preventing irritation of the parietal peritoneum and resulting in atypical or entirely absent physical signs (22). Consequently, the infection spreads silently along retroperitoneal pathways to the liver, transforming what is essentially an acute abdominal emergency into a case of fever of unknown origin. This serves as a strong warning to clinicians: the absence of abdominal pain must never be used as a criterion to exclude gastrointestinal perforation. When scrutinizing fever accompanied by a liver abscess, such silent perforations must be integrated into the core differential diagnosis (23).

### 3.2 Imaging: from sign recognition to a systemic diagnostic path

In this context, contrast-enhanced CT served not merely as a diagnostic tool but as a map revealing the pathological process. We summarize the key findings into a diagnostic Triad: (i) a liver abscess, (ii) a sinus tract connecting the duodenum to the abscess (direct or indirect sign), and (iii) inflammatory changes surrounding the duodenal wall. Recognizing this triad is critical. It is worth emphasizing that the foreign body itself is not always clearly visualized on imaging (24). Even when the object is obscured by encapsulation, indirect signs—such as haziness in the hepatoduodenal space or linear stranding—should be regarded as highly specific warning signals (25). The successful diagnosis in this case resulted from an active, systematic interpretation of the imaging rather than a passive search for the object itself. Therefore, actively tracing the migration track rather than passively waiting to spot the foreign body is key to improving diagnostic sensitivity (20, 26).

### 3.3 Laparoscopy: from minimally invasive technique to decision core

The role of laparoscopy in this case extended far beyond minimally invasive treatment. In scenarios where clinical suspicion is high but imaging is inconclusive, it acts as the final arbiter. Its value lies in its ability to execute a complete workflow simultaneously: exploration, definitive diagnosis, and treatment. This includes direct visualization of the foreign body, thorough debridement and drainage, and repair of the primary perforation (18). Compared to diagnostic antibiotic therapy or percutaneous drainage—which may delay treating the root cause—laparoscopy offers the most direct and curative solution, supporting its role as an effective and definitive diagnostic and therapeutic strategy.

### 3.4 An integrated diagnostic-therapeutic algorithm

This case suggests that a more actionable diagnostic algorithm can be established for clinical practice:

- Identify Atypical Clues: Recognize fever or elevated inflammatory markers combined with a liver abscess, specifically in the absence of biliary or portal vein infection evidence.
- Active Inquiry of Risk History: Specifically ask about the ingestion of sharp foreign bodies (fish bones, bone fragments) in the short term.
- Systematic Imaging Interpretation (The CT Triad): Move beyond reporting the abscess to assessing inflammatory changes in the hepatic-upper GI interspace and potential migration tracks.
- Early Curative Intervention: When foreign body etiology is highly suspected, prioritize integrated modalities (such as laparoscopic exploration) that allow for simultaneous diagnosis and treatment (27).

The core of this pathway is to transform the liver abscess from a final diagnosis into a starting clue. By locking onto the etiology through a mechanistic analysis of history and imaging, clinicians can reduce the time costs and potential complications associated with empirical anti-infective therapy. For patients with suspected foreign body-related liver abscesses, early radical intervention holds the promise of shortening the disease course and lowering the risk of recurrence (28).

### 3.5 Strengths and limitations

This case has several strengths. It highlights an important diagnostic pitfall: hepatic abscess secondary to migrated fish-bone perforation may present with isolated fever in the absence of abdominal pain, potentially delaying recognition. The report also demonstrates clear radiologic–surgical correlation, with computed tomography revealing a linear hyperdense foreign body and a suspected tract extending from the duodenum to the hepatic abscess, providing a useful diagnostic reference for clinicians. In addition, successful one-stage laparoscopic management with definitive source control and an uneventful short-term recovery supports the feasibility of minimally invasive treatment in selected patients.

This report also has limitations. As a single-case observation, the findings may not be generalizable. In addition, certain objective clinical parameters were not comprehensively documented, which may limit interpretability. Although short-term follow-up was reassuring, longer observation would be valuable to further confirm the durability of the outcome. Accumulation of additional similar cases is needed to better define optimal diagnostic and therapeutic strategies for this rare condition.

### 3.6 Patient perspective

In keeping with CARE guidelines, the patient perspective is presented below:

“At the early stage of my illness, I experienced recurrent fever of unknown cause without abdominal pain or obvious gastrointestinal discomfort, and therefore did not realize that the problem might originate from the digestive tract. Although I received antibiotic treatment for some time at a community clinic, the fever did not resolve, which left me feeling confused about the cause of my condition. After admission, I underwent a series of examinations. When I was first informed that an abnormal lesion had been found in my liver, I felt nervous and worried, particularly about the possibility of a malignant tumor. Subsequently, during further discussions and detailed questioning about my dietary history, I suddenly recalled that I had accidentally swallowed a fish bone before the onset of symptoms. Based on this information, the doctors explained the possible cause of my illness, which helped me better understand the origin of the disease and relieved my earlier concerns. When I was told that surgical treatment was necessary, I still experienced some anxiety. However, the operation proceeded smoothly, and my postoperative recovery was rapid. My body temperature returned to normal on the first day after surgery, and after gradually resuming oral intake, my physical condition and energy level improved noticeably. Throughout the entire treatment process, the careful care provided by the medical staff made me feel reassured.”

## Data Availability

The original contributions presented in the study are included in the article/supplementary material, further inquiries can be directed to the corresponding authors.
